# Mechanistic dissection of GRHL2 and PR transcriptional co-regulation in breast cells

**DOI:** 10.1371/journal.pgen.1012088

**Published:** 2026-03-17

**Authors:** Marleen T. Aarts, Anna Nordin, Claudio Cantù, Antonius L. van Boxtel, Renée van Amerongen

**Affiliations:** 1 Developmental, Stem Cell & Cancer Biology, Swammerdam Institute for Life Sciences, University of Amsterdam, Amsterdam, The Netherlands; 2 Wallenberg Centre for Molecular Medicine, Linköping University, Linköping, Sweden; 3 Department of Biomedical and Clinical Sciences, Division of Molecular Medicine and Virology, Faculty of Medicine and Health Sciences, Linköping University, Linköping, Sweden; 4 Science for Life Laboratory – SciLifeLab, Linköping University, Linköping, Sweden; Harvard Medical School, UNITED STATES OF AMERICA

## Abstract

Gene expression is controlled by complex transcriptional networks in which transcription factors and their cognate enhancer elements integrate developmental and environmental cues. The progesterone receptor (PR), a hormone-activated transcription factor, is essential for breast development and physiology, yet how it engages with the chromatin and lineage-specific cofactors remains unclear. Using an unbiased approach, we identify the epithelial transcription factor grainyhead-like 2 (GRHL2) as a key co-regulator of PR activity in hormone responsive breast cancer cells. We show that GRHL2 interacts with PR in a progesterone-independent manner. Upon progesterone stimulation, GRHL2 and PR are both recruited to distal enhancer elements of target genes. Furthermore, GRHL2- and PR-bound elements connect spatially through chromatin looping to regulate shared targets. These findings uncover a previously unrecognized mechanism by which GRHL2 and PR coordinate gene regulation through both chromatin binding and 3D genome architecture modification, positioning GRHL2 as a crucial modulator of steroid hormone receptor function.

## Author summary

Gene expression is tightly controlled by regulatory networks in which transcription factors integrate developmental and environmental cues. Hormones are one important class of such signals. In breast tissue, the hormone progesterone is essential for normal development and function. Progesterone binds to its receptor, the progesterone receptor, a DNA-binding protein that regulates the activity of progesterone-responsive genes. However, whether and how the progesterone receptor collaborates with lineage-specific cofactors to control gene expression has remained incompletely understood. In this study, we identified GRHL2 as a key co-regulator of the progesterone receptor in hormone-responsive breast cancer cells. We found that these two proteins interact even in the absence of hormone stimulation. Upon progesterone exposure, both proteins are recruited to regulatory regions that control shared target genes. Importantly, we demonstrate that these regulatory regions physically connect through long-range DNA looping, enabling coordinated gene expression. Together, our findings provide new insight into hormone-driven gene regulation and highlight GRHL2 as an important contributor to progesterone receptor function in breast cells.

## Introduction

Gene expression is tightly regulated by complex transcriptional networks, in which DNA-binding transcription factors (TFs) play a central role. These networks govern when and where genes are expressed by integrating cell-intrinsic developmental cues and extrinsic environmental signals [1]. TFs bind to distinct motifs in regulatory DNA elements to modulate transcriptional output [2–4]. Rather than acting alone, TFs operate in combinatorial assemblies with additional transcription factors, co-factors and chromatin remodelers. This layered regulatory architecture ensures that distinct gene expression programs are executed in a cell type– and context-specific manner, enabling the emergence of specialized cellular identities throughout development and their faithful maintenance in adult tissues [5,6].

Steroid hormone receptors represent a unique class of transcription factors, distinguished by their dual role as ligand-activated receptors and direct modulators of gene expression. Unlike many transcription factors with constitutive DNA binding activity, steroid receptor function is extrinsically regulated by the presence of specific hormones. Upon ligand binding, these receptors accumulate in the nucleus, where they dynamically associate with hormone response elements at the chromatin to regulate transcriptional programs [7].

This class of receptors includes the estrogen receptor (ER), which has its own unique DNA binding motif, and the 3-keto-steroid receptors, which share a consensus sequence: progesterone receptor (PR), mineralocorticoid receptor (MR), glucocorticoid receptor (GR) and androgen receptor (AR). Activated by ovarian hormones, ER and PR are key regulators of postnatal breast tissue development [8–10]. In this tissue, they are almost exclusively expressed in a subset of luminal cells, named the mature luminal or hormone-sensing cells. The majority of attention has been directed to resolving the signaling mechanism of ER, given its role (and targetability) in hormone-dependent breast cancer. The molecular mechanisms of PR signaling remain less well understood, but it regulates multiple cell intrinsic and paracrine signaling pathways. These pathways promote local proliferation and basement membrane restructuring [11] which in turn support ductal outgrowth, side branching and alveologenesis during pregnancy [10]. As such, PR is crucial for breast development and physiology and antiprogestins are actively explored from the perspective of breast cancer prevention and treatment [12].

Steroid receptors such as ER and PR do not act in isolation but function within complex regulatory networks involving additional associated proteins. Among these, pioneer factors, a class of transcription factors capable of modulating chromatin accessibility [13], have increasingly been recognized as critical co-regulators of steroid receptor activity, particularly in hormone-dependent cancers [14]. For instance, ER cooperates with pioneer factors such as FOXA1 and GATA3 to facilitate transcriptional activation of its target genes [15–20]. In contrast, the extent to which PR engages in similar interactions remains poorly understood. Grainyhead-like 2 (GRHL2) has recently emerged as a potential pioneer factor in hormone receptor-positive cancers, including breast cancer [21]. However, nearly all studies to date have focused on GRHL2 in the context of ER and estrogen signaling, leaving its role in PR- and progesterone-mediated regulation unexplored [22–26].

In this study, we identify and dissect the functional transcriptional interaction of PR and GRHL2, a member of the GRHL protein family. This family includes three evolutionarily conserved pioneer transcription factors (GRHL1, GRHL2, and GRHL3) with important roles in epithelia [27,28]. They share a highly conserved DNA-binding domain and display substantial structural homology [29,30]. Despite these similarities, the GRHL family members exhibit distinct spatiotemporal expression patterns and execute unique epithelial transcriptional programs that underpin their specific biological functions.

Here we combine genomic, transcriptomic and proteomic approaches to provide the first mechanistic dissection of the coordinate transcriptional activities of PR and GRHL2 in human breast cancer cells. We show that PR and GRHL2 can interact, independently of progesterone, and frequently co-occupy enhancer elements upon progesterone stimulation. Moreover, GRHL2 is required for the regulation of a set of PR target genes. Chromatin looping analysis further revealed how both shared (i.e., to the same DNA element) and distinct (i.e., to linearly separated DNA elements) PR and GRHL2 binding events can converge over long distances in 3D chromatin space to regulate common targets. Together, our findings uncover a new layer of PR gene regulation, shaped by GRHL2 and the 3D genome architecture.

## Results

### GRHL2 physically interacts with PR in a progesterone independent manner

**Fig 1:**
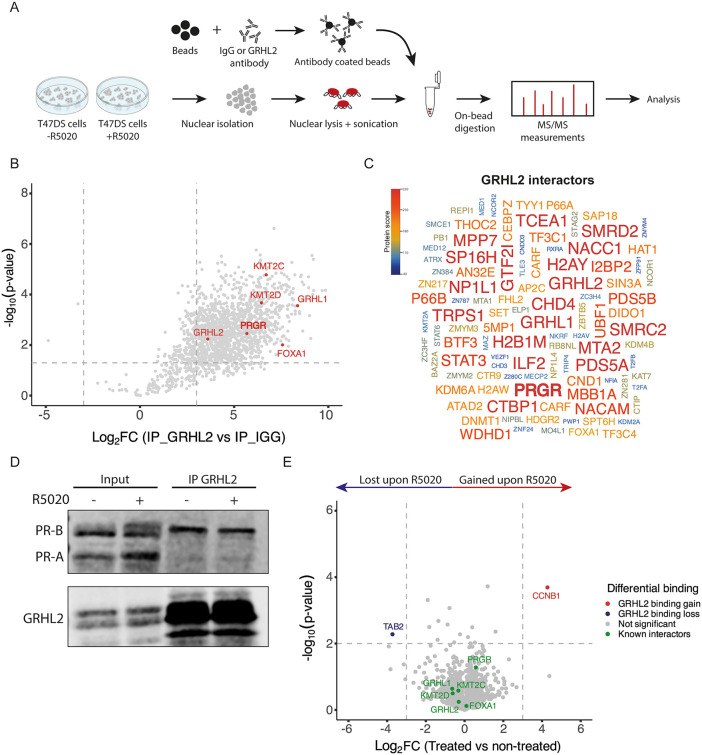
RIME analysis of GRHL2-associated proteins reveals GRHL2-PR interaction independent of progesterone. **A)** Schematic overview of the Rapid immunoprecipitation mass spectrometry of endogenous protein (RIME) [31] protocol used to identify GRHL2 interactors in hormone depleted and progesterone treated conditions. **B)** Volcano plot depicting the results of the GRHL2 RIME of hormone depleted conditions vs. the IgG control. Each grey dot represents a single protein identified in mass spectrometry. Grey dotted lines represent Log2FC (-3,3) and -Log10(p-value) cutoffs (0.05). A total of 2,352 proteins passed these criteria and were thus identified as a GRHL2 interactor. Highlighted dots in red are a few examples of previously reported GRHL2 interactors [18,27,32,33,35]. **C)** Word cloud presenting 103 GRHL2 interactors filtered as transcription factors or chromatin associated/modifying proteins according to the Panther Classification System [34]. The size and the color of the protein names represent the confidence of the identified interaction based on the MaxQuant protein identification scores (Andromeda score sum), reflecting the relative confidence of protein detection. **D)** Western blot of co-immunoprecipitation validation of the GRHL2 - PR interaction in hormone depleted and 4 hours 1 nM of the PR agonist R5020 stimulated conditions. Western blot shows GRHL2 and PR, isoform A and B, protein of input and immunoprecipitated samples. **E)** Volcano plot depicting the results of the GRHL2 RIME in hormone depleted vs. 4 hour 1 nM R5020 treated conditions. Each grey dot represents a single protein identified in mass spectrometry. Grey dotted lines represent Log2FC (-3,3) and -Log10(p-value) cutoffs (0.01). Only 2 proteins (1 lost, 1 gained) passed these criteria and were identified as a high-confidence progesterone dependent GRHL2 interactor. Highlighted dots in blue are proteins that lost GRHL2, highlighted dots in red are proteins that gained GRHL2 binding.

To identify nuclear interactors of GRHL2 in an unbiased manned, we performed Rapid immunoprecipitation mass spectrometry of endogenous protein (RIME) [31] in T47DS cells (**Fig 1A**). Under stripped-serum, non-hormone stimulated conditions we identified 2,352 GRHL2-associated proteins, including several previously reported nuclear interactors such as FOXA1 [18], KMT2C/D (MLL3/4) [32], GRHL1 [27,33] (**Fig 1B**). Of these 2,352 interactors, a total of 103 were annotated as transcription factors or chromatin-associated/modifying proteins, based on the Panther Classification System [34]. We ranked these proteins by the MaxQuant protein identification scores to identify and highlight high-confidence candidates (**Fig 1C**). Interestingly, several of the top-scoring interactors, such as CTBP1, MTA2, SMARCC2, SMARCD2, CHD3, and PHF6, are involved in chromatin accessibility and remodeling, which fits the established function of GRHL2 as a pioneering factor and modulator of chromatin accessibility [28] (**Fig 1C**).

Unexpectedly, PR emerged as one of the most robust GRHL2 interactors in these stripped-serum conditions, suggesting a hormone-independent association between the two factors **(Fig 1B and 1C)**. We validated this interaction by co-immunoprecipitation in both hormone-stripped vehicle-treated and hormone-stripped R5020 (PR-agonist)-treated T47DS cells, confirming that the GRHL2–PR interaction remained unaffected by progesterone stimulation (**Fig 1D**). Interestingly, we find that GRHL2 mainly binds to the full-length and transcriptionally active isoform of PR, PR-B, but not to PR-A (**Fig 1D**).

Since the GRHL2-PR interaction is not dependent on the presence of progesterone, we wondered if and how progesterone altered the GRHL2 interactome. Therefore, we also performed the GRHL2 RIME in T47DS cells following 4 hour treatment with the synthetic progestin R5020 (**Fig 1E**). Comparison with the untreated condition revealed that only few interactions were affected. For only one protein, TAB2, the interaction with GRHL2 was lost upon progesterone stimulation. Conversely, again only one protein, CCNB1, was recruited only in the presence of hormone (**Fig 1E**). These differentially interacting proteins were not classified as transcription factors or chromatin regulators, and no direct relation to progesterone or PR has thus far been reported in the literature, although both have been implicated in breast cancer [36,37]. Since the GRHL2-PR interaction itself has also not been functionally characterized, we focused our subsequent analyses on this potential transcriptional partnership.

### Global mapping of GRHL2 and PR DNA binding reveals co-occupancy at enhancer sites

**Fig 2:**
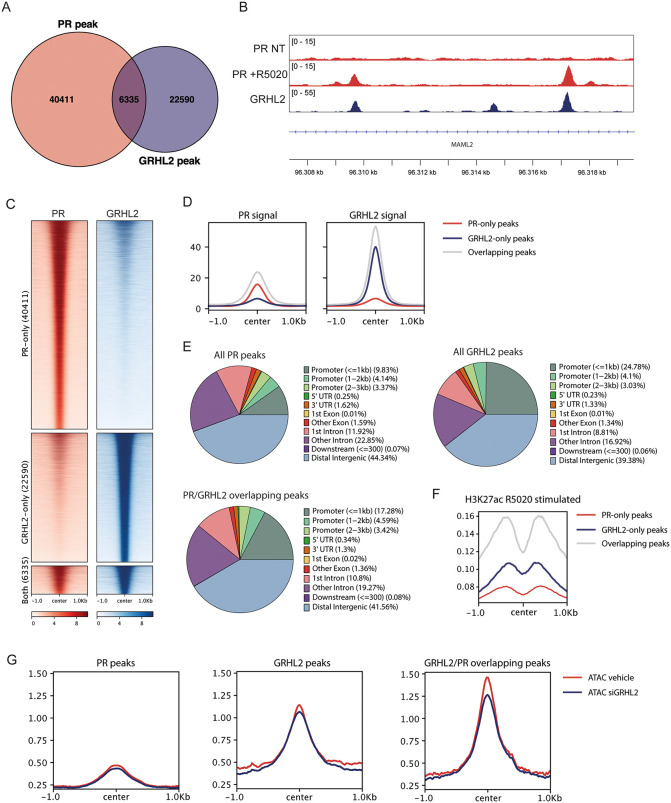
GRHL2 and PR co-bind regulatory regions across the genome. **A)** Venn diagram showing the overlap between non-treated GRHL2 [23] Chip-seq and 1 nM R5020 stimulated PR [38] ChIP-seq peaks. Peaks were considered overlapping if they overlapped by at least 1 bp. **B)** A representative example of two PR and GRHL2 overlapping peaks. Data was visualized in the IGV browser [39]. NT = non-treated. **C)** Heatmaps showing binding ChIP-seq signal of non-treated GRHL2 [23] and 1 nM R5020 stimulated PR [38] at PR-only, GRHL2-only and GRHL2-PR co-occupied genomic regions. **D)** Signal intensity plots showing the cumulative ChIP-seq signal of GRHL2 [23] and 1 nM R5020 stimulated PR [38] at the PR-only, GRHL2-only and GRHL2-PR co-occupied genomic sites. **E)** Genomic annotation of all PR, all GRHL2 or GRHL2-PR shared peaks by ChIPseeker [40,41]. Sites were annotated to 5’UTR, promotors, 1st exon, other exons, 1st intron, other introns, 3’UTR introns, distal intergenic regions and downstream. Promotor regions were divided into three subcategories defined as < 1 kb from the TSS, 1-2 kb from the TSS or 2-3 kb from the TSS. **F)** Signal intensity plots showing the cumulative ChIP-seq signal of H3K27ac [42] in 30 minutes R5020 stimulated T47D cells at the PR-only, GRHL2-only and GRHL2-PR co-occupied genomic sites. **G)** Signal intensity plots showing the cumulative non-treated ATAC signal [43] of siControl and siGRHL2 T47D cells at the PR-only, GRHL2-only and GRHL2-PR co-occupied genomic sites.

To determine whether the GRHL2–PR interaction occurs at shared genomic loci, we re-analyzed two previously published ChIP-seq datasets for GRHL2 [23] and PR [38] in T47D cells. Of note, the GRHL2 dataset was generated under stripped-serum conditions, thus representing GRHL2 binding under estrogen and progesterone depleted conditions. The PR dataset was also generated under stripped-serum conditions, but here the cells were simultaneously stimulated with 1 nM R5020. In this analysis, we identified 46,746 PR and 28,925 GRHL2 peaks. Of these, 6,335 sites were overlapping, representing 13.5% of the PR and 21.9% of the total GRHL2 binding events (**Fig 2A**). One example of such a GRHL2-PR shared region is shown (**Fig 2B**). Heatmap visualization of PR and GRHL2 binding at PR-only, GRHL2-only and GRHL2-PR overlapping sites revealed that the strongest PR and GRHL2 signal is concentrated at overlapping regions, although some GRHL2 signal is also detected at the strongest PR peaks and vice versa (**Fig 2C-D**). The observation that GRHL2 and PR signals co-occur beyond the defined overlapping peaks suggests that their functional cooperation likely extends beyond the stringently thresholded sites identified in our analysis (Fig 2C-D). Genomic annotation of the PR and GRHL2 ChIP-seq peaks showed that the majority of the PR (~66%) and GRHL2 (~57%) sites, including the overlapping PR-GRHL2 (~61%) sites are located at enhancers in distal intergenic or intronic regions (**Fig 2E**). Notably, GRHL2-PR overlapping regions corresponded to active enhancers marked by H3K27ac under R5020 stimulated conditions (**Fig 2F**). GRHL2 peaks were more frequently located within 1 kb of a transcription start site (TSS) than PR containing peaks (~25% of all GRHL2 peaks and ~17% of GRHL2-PR shared sites, compared to <10% of all PR peaks) (**Fig 2E**). Moreover, GRHL2–PR overlapping regions were more often associated with accessible chromatin than PR-only or GRHL2-only sites, as determined by ATAC-seq (**Fig 2G**). Consistently, siRNA-mediated GRHL2 knockdown altered chromatin accessibility at GRHL2–PR shared sites, suggesting that GRHL2 contributes to maintaining accessibility at these regions (**Fig 2G**). Motif analysis confirmed strong enrichment for PR and GRHL2 motifs in both their individual and overlapping peaks (**S1A Fig**). Moreover, PR or GRHL2 bound, as well as GRHL2-PR overlapping regions were enriched for FOXA1 motifs. In addition, TEAD motifs were specifically enriched in GRHL2 bound regions, including GRHL2-PR overlapping sites. This suggests that both FOXA1 and TEAD act on similar regulatory regions as GRHL2 and PR in breast tissue (**S1A Fig**).

**Fig 3:**
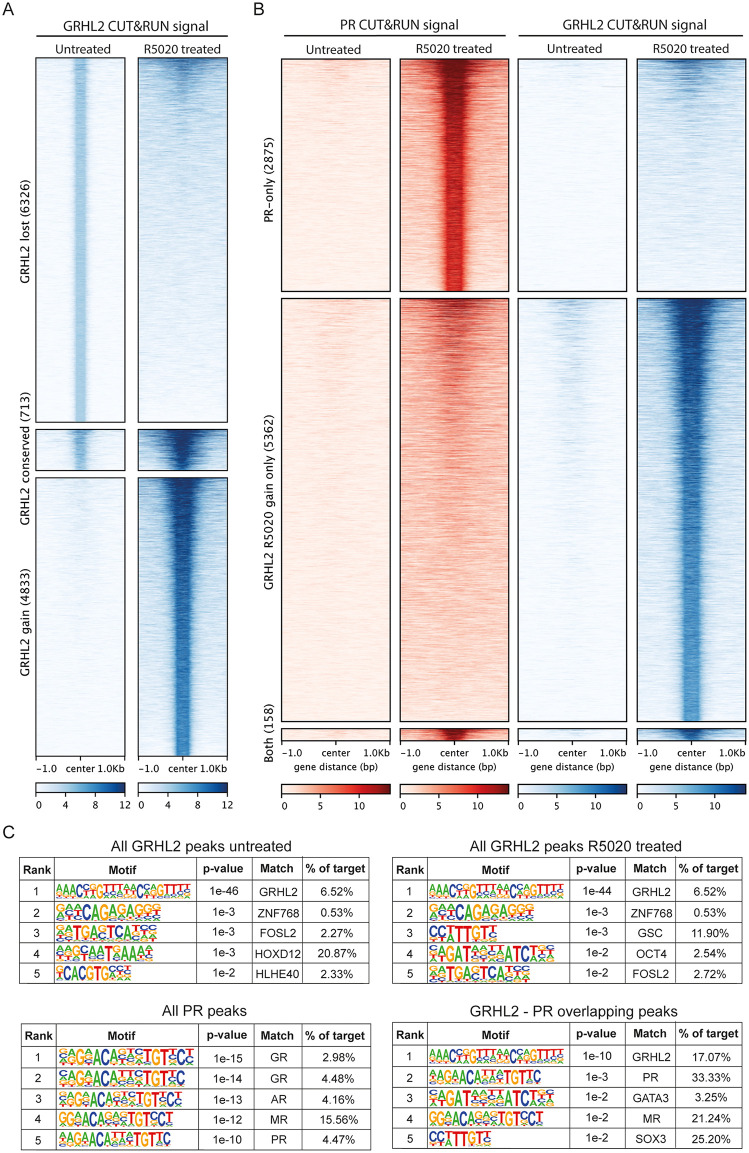
CUT&RUN reveals GRHL2 redistribution upon progesterone stimulation. **A)** Heatmaps showing CUT&RUN GRHL2 signal in untreated and 4 hour 1 nM R5020 stimulated conditions at sites identified as GRHL2 bound in hormone depleted (GRHL2 lost), GRHL2 bound in hormone depleted and R5020 stimulated conditions (GRHL2 conserved), or GRHL2 bound in R5020 stimulated conditions (GRHL2 gain). **B)** Heatmaps showing CUT&RUN signal of GRHL2 in untreated and 4 hour 1 nM R5020 stimulated conditions, and untreated and 4 hour 1 nM R5020 stimulated PR signal at PR-only, GRHL2 bound in R5020 stimulated conditions and GRHL2 R5020 stimulated and PR co-occupied CUT&RUN peaks. **C)** Top 5 transcription factor motifs that were determined as enriched in all untreated GRHL2 peaks, all R5020 treated GRHL2 peaks, all R5020 treated PR CUT&RUN peaks, and all GRHL2 R5020 treated and PR co-occupied CUT&RUN peaks as determined by HOMER motif analysis [44].

We next asked if and how progesterone would modulate chromatin binding of GRHL2. To obtain more insight in such binding dynamics, we performed CUT&RUN analysis for GRHL2 and PR in hormone deprived T47DS cells under both unstimulated and progesterone-stimulated conditions. In the absence of hormone stimulation and in stripped-serum conditions, we identified 7,039 GRHL2 peaks, whereas 5,520 GRHL2 peaks were identified following 4 hours of R5020 treatment (**Fig 3A**). Only 713 of these sites overlapped. Moreover, the GRHL2 signal significantly increases upon R5020 stimulation, indicating that GRHL2 chromatin binding is both redistributed and strengthened upon R5020 stimulation (**Fig 3A**). This effect is unlikely to be fully explained by a mere increase in GRHL2 protein levels, as 4 hour R5020 treatment elevated GRHL2 protein levels only slightly, with at most a 20% (and statistically insignificant) increase in protein abundance (**S2A-B Fig**). For PR, we identified 3,033 peaks after 4 hours of R5020 stimulation. Overlapping the GRHL2 and PR peaks revealed that GRHL2 and PR binding sites only overlap under R5020-treated condition (158 sites, **Figs 3B**, **S1B-S1C**). Motif analysis of these sites confirmed enrichment for both PR and GRHL2 motifs, as expected (**Fig 3C**). Given that PR and GRHL2 biochemically interact in both the absence and presence of progesterone (**Fig 1**), this suggests that PR activation facilitates GRHL2 recruitment to the progesterone responsive chromatin elements.

Plotting our CUT&RUN signal on the GRHL2 bound and GRHL2-PR shared ChIP-seq peaks identified in Fig 2 also confirms an R5020-dependent increase in GRHL2 binding at these locations (**S1D Fig**). The CUT&RUN GRHL2 signal observed under untreated conditions, while reduced in intensity, also shows substantial overlap with GRHL2 bound and GRHL2–PR shared ChIP-seq peaks. Of note, our PR CUT&RUN signal under R5020 stimulated conditions is not only enriched at PR bound and GRHL2-PR shared ChIP-seq peaks, but also at peaks identified as GRHL2 only ChIP-seq peaks. These combined observations suggest that for some reason our R5020-stimulated GRHL2 CUT&RUN condition in T47DS mirrors the previously reported unstimulated GRHL2 ChIP-seq data in T47D.

Taken together, PR and GRHL2 can bind to the DNA independently, but can also overlap at enhancer regions. Moreover, GRHL2 chromatin binding is strengthened and redistributed upon progesterone stimulation. This supports a model in which PR and GRHL2 cooperate to orchestrate hormone-dependent transcriptional regulation.

### GRHL2 and PR co-regulate a subset of genes involved in breast development

**Fig 4:**
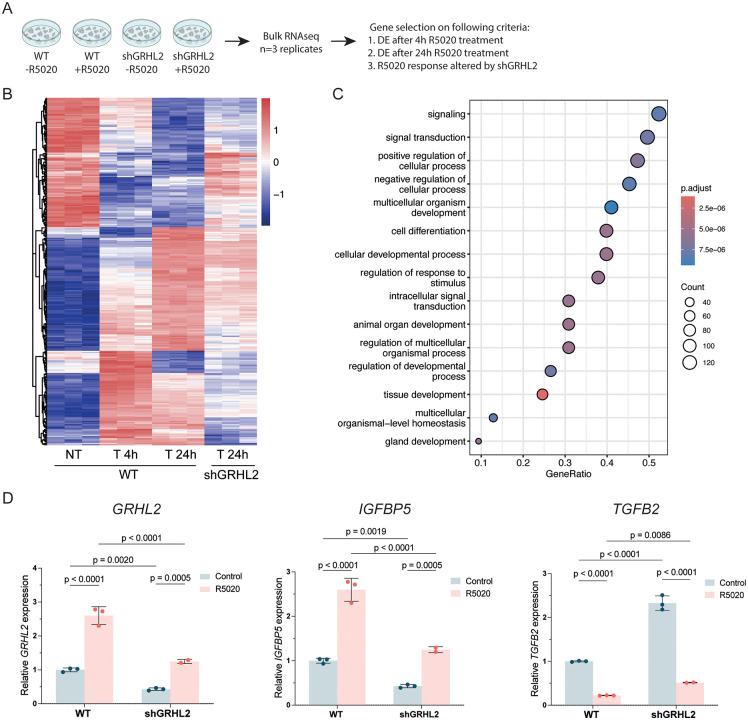
GRHL2 and PR co-regulate target genes involved in breast development. **A)** Schematic overview of the bulk RNA-seq process on wildtype (WT) T47DS cells and shRNA mediated *GRHL2* knockdown (shGRHL2) T47DS cells stimulated with 1 nM R5020 for 24 hours and the criteria used to select GRHL2 and PR co-regulated genes. **B)** Heatmap of bulk RNA-seq data from WT and shGRHL2 T47DS cells stimulated with 1 nM R5020 for 4 or 24 hours. Heatmap shows unsupervised clustering and expression changes of 298 genes that were selected based on the criteria listed in **(A)**. Expression values are depicted as z-scores for n = 3 replicates. NT = non-treated stripped-serum condition, T = treated condition. **C)** Dot plot showing Go term enrichment analysis on the 298 selected GRHL2 and PR co-regulated genes. Enrichment analysis and figure generation was done using Clusterprofiler [49]. **D)** Bar graphs of qRT-PCR data, depicting the mean relative expression of *GRHL2, IGFBP5* and *TGFB2* after 4 hours of 1 nM R5020 treatment in WT or shGRHL2 T47DS cells. Reference gene: *YWHAZ*. Datapoints: individual values for n = 2/3 biological replicates depicted as mean fold change normalized to control. P-values were calculated using a two-way ANOVA followed by an Uncorrected Fisher’s Least Significant Difference test.

Next, we sought to identify transcriptional programs regulated by GRHL2 and PR. To this end, we generated T47DS cells with a stable shRNA-mediated knockdown of *GRHL2* (**S2A-B Fig**) and performed RNA-sequencing on wild-type and *GRHL2* knockdown T47DS cells treated with either vehicle, R5020 for 4 or R5020 for 24 hours. Principal component analysis (PCA) showed that 97% of the variance in gene expression was explained by the R5020 stimulation (PC1) or *GRHL2* knockdown (PC2), confirming robust transcriptional responses to our experimental conditions (**S2C Fig**). To identify direct and sustained GRHL2 and PR co-regulated genes, we generated a stringent selection based on three criteria: 1) statistically significant differential gene expression after 4 hours of R5020 treatment, 2) statistically significant differential gene expression after 24 hours of R5020 treatment, and 3) a significantly altered transcriptional response to R5020 upon GRHL2 depletion (**Fig 4A**). Applying these criteria, we identified 298 genes regulated by both PR and GRHL2 (**Fig 4B**). Gene Ontology enrichment analysis identified genes involved in signal transduction and cellular processes as well as several developmental processes including gland development (**Fig 4C**). We validated the expression changes by qRT-PCR for selected target genes, including *GRHL2* itself, *IGFBP5* and *TGFB2* (**Fig 4D**). Together, these results reveal a large number of genes that are jointly regulated by GRHL2 and PR, confirming their co-regulatory role in hormone responsive breast cancer cells.

We performed a first exploratory analysis to assess the potential clinical relevance of the 298 genes co-regulated by GRHL2 and PR. To this end, clinical and transcriptomic data from the METABRIC (Molecular Taxonomy of Breast Cancer International Consortium) cohort, comprising 1,980 primary breast tumors, were downloaded via cBioPortal [45–48]. Of these, 940 tumors were classified as PR-negative and 1,040 as PR-positive. Indeed, the assigned PR status corresponded with PGR mRNA expression levels (**S3A Fig**). Importantly, GRHL2 expression did not differ between PR-negative and PR-positive tumors (**S3B Fig**).

Subsequently, we calculated a composite GRHL2/PR signature score and stratified tumors into high- and low-signature groups based on the median. Kaplan–Meier analysis showed significantly reduced overall survival in patients with high signature expression (**S3C Fig**), and multivariable Cox regression adjusting for age and tumor stage confirmed this association (HR = 1.055, 95% CI 1.030–1.080, *p* = 1.1 × 10 ⁻ ⁵). Counterintuitively, PR-positive tumors had significantly lower signature scores than PR-negative tumors (**S3D Fig**). Since low signature scores were associated with improved survival, we tested whether the prognostic impact of the signature differed by PR status using a multivariable Cox model with an interaction term. The interaction was not significant (HR = 1.038, 95% CI 0.976–1.103, *p* = 0.24), indicating that higher signature expression is comparably associated with worse outcome in both PR-positive and PR-negative tumors. From this we tentatively conclude that while the GRHL2/PR signature relates to poorer prognosis, this is independent of the PR status. Nonetheless, it is important to note that this analysis is exploratory. Functional PR signaling in primary tumors is difficult to assess directly, and cell line models may not fully recapitulate tumor biology. Therefore, further validation and stratified analyses will be required to further asses the prognostic value of the PR/GRHL2 score in breast cancer patients.

### Chromatin looping connects GRHL2 and PR sites to distal target genes

Despite significant advances in our understanding of 3D genome organization, it remains challenging to functionally link enhancers to their target genes [50]. In most studies, enhancers are assigned to the nearest gene, despite that fact that that 33–73% of enhancers do not necessarily regulate their closest gene [51–53]. This is important since PR, as well as many other transcription factors, frequently bind distal enhancers located between 30 kb and 1 Mb away from their true associated TSSs [54, 55]. High-resolution chromatin conformation capture techniques—such as Hi-C, 5C, ChIA-PET, and HiChIP—enable the identification of long-range chromatin interactions that are essential for transcriptional regulation by such factors [50].

**Fig 5:**
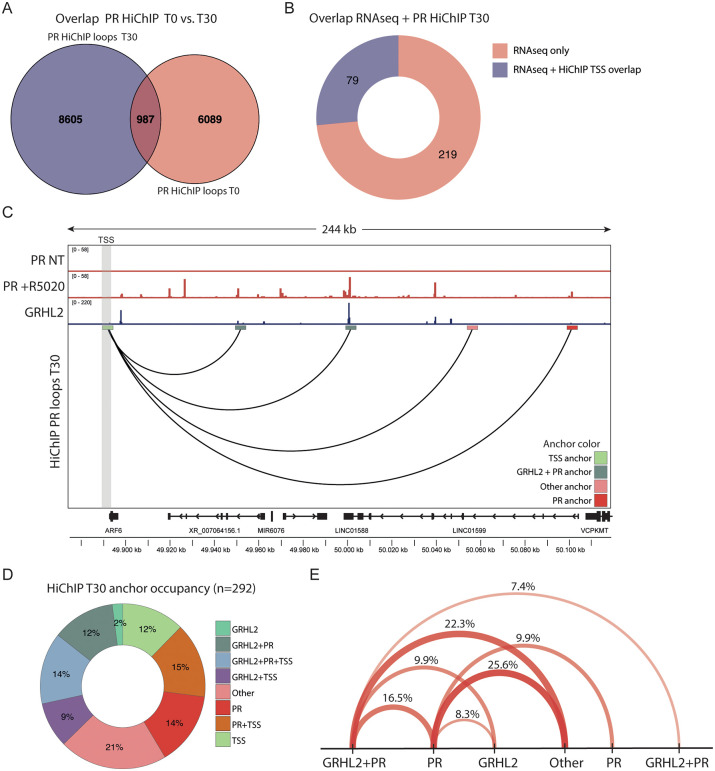
Integrative analysis of HiChIP, ChIP-seq, and RNA-seq reveals functional GRHL2–PR loops. **A)** Venn diagram showing the overlap between PR HiChIP loops in the non-treated (T0) condition and the 30 minutes 10 nM R5020 treated condition (T30). Replotting of data (i.e., number of called loops from Zaurin *et* al. [38] **B)** Donut chart exhibiting the proportion of transcription start sites (TSSs) of the GRHL2 and PR regulated genes that overlaps with an anchor from a previously published PR HiChIP [38]. Of the 298 GRHL2 and PR regulated genes identified by RNA-seq, 79 genes have a TSS that overlaps with a HiChIP anchor. The TSS of the remaining 219 genes does not overlap with a HiChIP anchor. **C)** Visual representation of how GRHL2 and PR peaks converge on the *ARF6* gene promotor by chromatin looping, showing PR ChIP-seq [38] peaks, GRHL2 ChIP-seq [23] peaks and PR HiChIP loops [38] directly anchoring to the GRHL2/PR regulated TSS. Anchors were colored by GRHL2 and PR occupancy as depicted in the legend at the lower right corner, anchors without GRHL2 or PR ChIP-seq signal or an annotated TSS were defined as ‘Other’. The gene TSS is highlighted with the grey bar. Data was visualized in the IGV browser [39]. **D)** Donut chart illustrating the HiChIP [38] anchor occupancy categorized as overlapping with GRHL2 peak, PR peak, TSS, combinations of these three or other. The 292 included anchors (two for each of the 146 loops) were associated with the TSS of the 79 GRHL2 and PR regulated genes that overlap with a HiChIP [38] anchor. **E)** Visual representation of the frequency of anchors defined as overlapping with GRHL2, PR, GRHL2 + PR ChIP-seq peaks or other at opposing anchor pairs of the 146 selected HiChIP [38] loops. The 121 loops were selected to have a PR ChIP-seq peak on at least one of the anchors. Color and thickness of the line represent the occurrence frequency.

With this in mind, we sought to physically link the genomic GRHL2 and PR sites (as identified by ChIP-seq, **Fig 2**), to the genes they regulate (as identified by RNA-seq, **Fig 4**). We reasoned that if a GRHL2 or PR site was involved in the regulation of a specific gene, it should loop to the TSSs of this gene. To test this hypothesis, we used a previously published PR HiChIP dataset obtained using T47D cells [38]. HiChIP is an extension of Hi-C that captures protein-specific chromatin loops by enriching for DNA interactions bound by a transcription factor or histone modification of interest. Each chromatin loop contains two anchors, one on either end, representing interacting DNA regions brought into proximity by 3D chromatin looping. The PR HiChIP captured 7,076 chromatin loops involving PR without stimulation and 9,591 chromatin loops involving PR at one or both anchors after 30 minutes of R5020 stimulation (**Fig 5A**) Only 987 of those loops overlapped, showing substantial rewiring of the PR mediated chromatin loops upon R5020 stimulation, as early as 30 minutes after stimulation (**Fig 5A**).

Next, we integrated the PR HiChIP data with the GRHL2 and PR ChIP-seq and RNA-seq data. Here, we used the previously published ChIP-seq data instead of our newly generated CUT&RUN dataset, as HiChIP is more methodologically similar to ChIP, and therefore more likely to integrate effectively. First, we took all 298 GRHL2 – PR co-regulated genes from our RNA-seq analysis and assessed if their TSSs overlapped with an anchor of at least one of the HiChIP loops. Using this approach, we identified 79 genes that are transcriptionally regulated by GRHL2 and PR and that also harbor a PR HiChIP loop anchor at or near their TSS (**Fig 5B**). In total, we identified 146 loops directly connecting to the 79 GRHL2 and PR regulated genes (Fig 5B-5C). These loops extended up to 500kb, with the majority spanning 50–100kb (**S4A Fig**). We subsequently wondered if and to what extent GRHL2 and PR could be detected at the anchors of these 146 loops. Therefore, we aligned the GRHL2 and PR ChIP-seq data to assess their occupancy at each anchor. For all 146 loops, we categorized the associated 292 anchors according to the presence of a GRHL2 and/or PR ChIP-seq peak and/or a TSS (**Fig 5C**, 5D). Among the annotated anchors, 26% were shared between GRHL2 and PR (~two-fold enrichment compared to the entire ChIP-seq dataset, **Fig 2B**), of which 14% was located at or near a TSS.

Another 29% only contained a PR peak, of which 15% located at or near a TSS, while 11% only contained a GRHL2 peak, of which 2% located at or near a TSS (**Fig 5D**). Notably, 21% of loop anchors, fell into the ‘Other’ category, meaning that they lacked either GRHL2, PR, or a TSS annotation (**Fig 5D**). We hypothesize that these anchors are likely occupied by additional transcriptional regulators involved in the joint control of GRHL2 and PR target genes. To explore this possibility, we performed motif enrichment analysis specifically at these sites (**S4B Fig**). This analysis revealed no transcription factors that have specifically been linked to the breast before. However, we observed significant enrichment of STAT3, a factor that was also observed in the GRHL2 RIME (Fig 1C), indicating that STAT factors potentially contribute to the 3D regulatory context in which GRHL2 and PR exert their function (**S4B Fig**).

To further investigate the cooperative function of GRHL2 and PR in gene regulation, we analyzed their occupancy at opposing anchors of the PR HiChIP loops (**Fig 5E**). Here, we only included loops that have a PR ChIP-seq peak on at least one of the two anchors, resulting in a selection of 121 loops. Of these, ~ 56% contain at least one anchor that contains both GRHL2 and PR ChIP-seq peaks, indicating that the most prevalent mode of GRHL2–PR–mediated gene regulation potentially involves co-binding of GRHL2 and PR to at least one regulatory element (**Fig 5E**). Nonetheless, all other combinations of GRHL2 and PR occupancy were also observed, suggesting that distinct GRHL2- and PR-bound elements can converge through 3D chromatin architecture to jointly regulate gene expression (**Figs 5E**, S4C). Moreover, some PR, GRHL2 or GRHL2-PR overlapping peaks not only establish direct connections with gene promoters but also interact with additional GRHL2 and PR (as well as other transcription factor) binding sites (**S4C Fig**).

### An integrative model for GRHL2 and PR transcriptional coregulation

CTCF- and Cohesin-mediated looping is well established as a key mechanism facilitating long-range enhancer–promoter interactions [56]. In our RIME, we identified GRHL2 to be associated with multiple Cohesin complex components and related regulatory factors (**S5A Fig**). Furthermore, publicly available ChIP-seq datasets for CTCF [42] and RAD21 [42] in non-treated and 30 minutes R5020 treated conditions revealed that while CTCF binding is relatively stable, RAD21 binding to chromatin is highly enriched upon R5020 treatment (**S5B-C Fig**). Since TAD boundaries are relatively stable irrespective of R5020 presence [57], we hypothesize that a subset of GRHL2/PR/TSS interactions are mediated by CTCF/Cohesin mediated looping, thus playing a role in the induced spatial proximity between GRHL2–PR enhancers and their target promoters. We speculate that the RAD21/Cohesin complex may possibly be recruited to the chromatin together with GRHL2 in the presence of progesterone.

**Fig 6:**
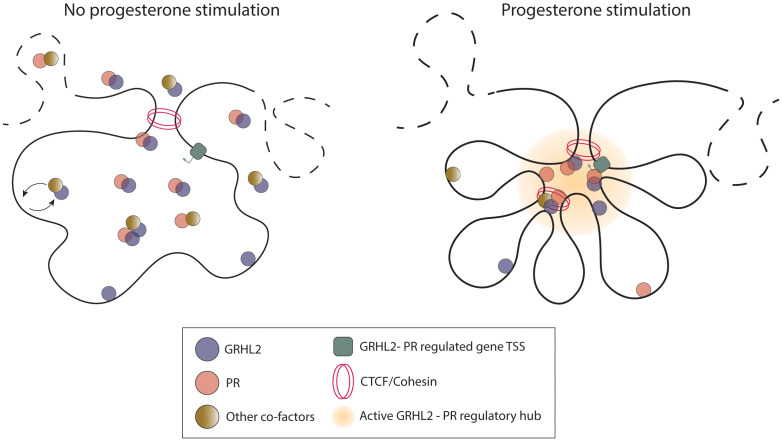
A model illustrating the transcriptional regulation of a GRHL2–PR co-regulated target gene. Model for the regulatory mechanism of GRHL2 and PR at a given co-regulated target gene. In absence of hormone (left), GRHL2 and PR form a complex, GRHL2 is dynamically bound to regions not associated with GRHL2 – PR target genes, as well as dynamically and at much lower levels, to shared GRHL2/PR sites, PR is largely not chromatin bound. As a result, GRHL2 and PR do not interact with their target promotors. Progesterone stimulation (right) triggers the recruitment of GRHL2 and PR to enhancers and the formation of both CTCF/Cohesin-dependent and independent chromatin loops to connect distal elements co- or individually bound by GRHL2, PR or co-factors to their target genes.

Taking all data together, we propose a model for genes co-regulated by GRHL2 and PR in which, in the absence of hormonal stimulation, GRHL2 and PR are already present in the nucleus and capable of physically interacting with each other (**Fig 6**, **left**). Under these conditions, PR is largely unbound to its genomic target sites, whereas GRHL2 is bound to regions not necessarily associated with GRHL2 and PR co-regulated target genes, However, it is likely already dynamically, and at much lower levels, binding to shared GRHL2/PR sites (**Fig 6**, **left**). Most genomic target sites and regulatory elements bound by either or both factors are not yet in the proximity of the promoter of their respective target gene (**Fig 6**, **left**). In the presence of progesterone or, in an experimental setting, upon R5020 stimulation, the levels of nuclear GRHL2 and PR subtly increase, and transcriptionally active PR is recruited to GRHL2/PR shared and PR only binding sites. At the same time, GRHL2 chromatin binding is strengthened and reorganized upon progesterone stimulation.

In addition to binding each other, GRHL2 and PR also associate with various co-factors. This results in locally high concentrations of GRHL2 and PR, facilitated by both CTCF/Cohesin-dependent and -independent loops to connect distal enhancers to target promoters (**Fig 6**, **right**). While more than half of all PR-looped enhancers are co-bound by both GRHL2 and PR on at least one anchor, distinct GRHL2- or PR-bound elements also converge on promoters (**Fig 6 right**). Moreover, multiple enhancer sites—bound by GRHL2 and/or PR as well as other transcriptional co-factors—are brought in close proximity with gene promoters through direct and potentially by indirect looping, enabling efficient transcription of genes involved in progesterone-driven breast tissue development and physiology (**Fig 6**, **right**).

## Discussion

In this study, we provide a mechanistic framework for the coordinated transcriptional regulation by the pioneering transcription factor GRHL2 and the steroid hormone receptor PR. By integrating proteomic, transcriptomic, and genomic analyses in T47D and T47DS cells in both unstimulated and R5020 stimulated conditions, we propose a model for GRHL2 and PR co-regulated target genes in which, in the absence of hormone, GRHL2 and PR form a pre-assembled nuclear complex that is associated with other chromatin modifying factors (Figs 1A-D and Fig 6, **left**). Upon progesterone stimulation, GRHL2 and PR become enriched at specific enhancers, initiating hormone-dependent chromatin looping that brings distal regulatory elements (either co-bound by GRHL2 and PR or individually bound by either factor or co-factors) into contact with their target gene promoters to activate transcriptional programs essential for breast development and physiology (**Fig 6**, **right**). Thus, while the overall nuclear GRHL2 protein interactome does not undergo major changes upon hormone stimulation (**Fig 1E**), these findings uncover a dynamic, hormone-responsive regulatory architecture that enables precise spatiotemporal control of gene expression in breast cells.

Using RIME and co-immunoprecipitation, we identified PR as a progesterone-independent interactor of GRHL2 under serum-stripped conditions. Although RIME involves a nuclear enrichment step that favors chromatin-associated proteins, the immunoprecipitation is performed on nuclear lysates and therefore captures both chromatin-bound and soluble nuclear complexes. Consequently, the observed PR–GRHL2 interaction in the absence of ligand likely reflects nuclear complexes that are not stably bound to chromatin. This is consistent with the known requirement of ligand for stable PR–DNA binding. We propose that PR and GRHL2 may form pre-existing complexes within the nucleoplasm, which can rapidly associate with chromatin upon ligand stimulation. Consistent with this idea, GRHL2 binding is strengthened and redistributed upon R5020 treatment, suggesting that pre-formed PR–GRHL2 complexes may facilitate the swift recruitment of GRHL2 to progesterone-responsive genomic regions.

The GRHL2–PR interaction can also be found in data reported in a study that performed PR RIME in both MCF7 and T47D cells [35], but here it appears as being progesterone-dependent. Although both studies detect an interaction between PR and GRHL2, our findings thus differ in the hormone-dependency of this association. The most likely explanation is that Mohammed *et al*. [35] employed a non-treated condition involving full-serum medium, which contains endogenous estrogen and progesterone and can mimic physiological hormone stimulation. In contrast, our non-stimulated sample was obtained under stripped-serum conditions. GRHL2 has previously been shown to associate with other hormone receptors, namely ER [23,25,58] and AR [59]. Specifically, GRHL2 enhances both ER- and AR-dependent binding and transcriptional activity [25,59]. In prostate cancer cells, GRHL2 itself is an AR target gene [59], similar to our observation that GRHL2 is a PR target in breast cancer cells [60]. Because GRHL2 functions in estrogen-, androgen-, and progesterone-signaling pathways, in cells where multiple hormone receptors may be expressed and active at any one time, GRHL2 binding may be dynamically distributed. The mechanisms governing this distribution remain unclear. Notably, we find that in T47DS cells and in the absence of both estrogen and progesterone, GRHL2 preferentially associates with PR, and that this interaction is maintained—or potentially enhanced—upon progesterone stimulation. Combining this finding with the observations of the previous study, we hypothesize that, in the presence of estrogen, either alone or together with low levels of progesterone, GRHL2 may preferentially associate with ER rather than PR. Together, this raises important questions regarding how GRHL2’s DNA binding activity and interactome are regulated under fluctuating hormonal conditions, and how such modulation contributes to transcriptional programs in hormone-responsive cells.

In this study, we only briefly explored which additional transcription factors may participate in GRHL2–PR–mediated gene regulation. Nevertheless, several transcription factors and transcription factor families were identified in both our RIME and motif analyses of PR-, GRHL2-, and GRHL2–PR–shared binding sites. For example, FOXA1 emerged as one of the most strongly enriched motifs in genome-wide analyses of GRHL2-, PR-, and GRHL2–PR shared regions (**S1A Fig**), as well as among the enriched motifs in our PR CUT&RUN data, consistent with its established central role in mature luminal cells [43,61–63]. Interestingly, FOXA1 depletion has been reported to increase PR binding at enhancer sites [64], suggesting that FOXA1 may, in some contexts, compete with PR for enhancer occupancy. Together, these observations suggest that while GRHL2 and FOXA1 may cooperate in certain settings, GRHL2–PR co-regulatory activity likely occurs largely independently of FOXA1. Similarly, GATA3 and TRPS1 were detected in our RIME analysis, and GATA motifs were enriched in PR-only, GRHL2-only, and GRHL2–PR–shared regions in the ChIP-seq data. Given that GATA3 has been linked to ER expression and function [19,20], it may play a role analogous to GRHL2 in coordinating ER/PR activity. Lastly, STAT1, STAT3, and STAT6 were identified as GRHL2 interactors, and STAT binding motifs were enriched across PR-only, GRHL2-only, and GRHL2–PR–overlapping sites in the ChIP-seq data. IL/STAT signaling has been previously associated with both ER and PR pathways [65,66], but to our knowledge, our study represents the first evidence linking GRHL2 to STAT signaling, warranting further investigation of their potential functional interplay.

Our CUT&RUN analysis showed that GRHL2 chromatin binding is redistributed and increased upon progesterone presence. This has not been reported before, but it is in line with GRHL2 being a direct PR target [60] and with prior work showing that estrogen treatment leads to a genome-wide increase in GRHL2 binding [58]. Interestingly, we find that GRHL2 CUT&RUN peaks detected following R5020 treatment, but not those from untreated conditions, overlap with GRHL2 sites identified by ChIP-seq under none-hormone stimulated conditions (**S1D Fig**). This discrepancy may be explained by residual hormone activity during the original ChIP-seq experiment, or by methodological differences: unlike ChIP-seq, CUT&RUN does not involve cross-linking and is therefore more likely to capture stable DNA–protein interactions. Transient, weaker or indirect interactions, such as those involving dynamic transcription factors like GRHL2 and PR, may only be detectable by CUT&RUN when stabilized (e.g., by hormonal treatment), whereas ChIP-seq may capture these interactions more readily. These methodological and biological differences may also explain why the number of GRHL2, and PR peaks identified in our CUT&RUN data is nearly two orders of magnitude lower than in the reanalyzed ChIP-seq dataset (see **Figs 3B** vs. **2B**).

Ultimately, it remains challenging to determine the precise function of individual enhancers and to accurately link them to their target genes. This difficulty largely stems from the location of enhancers, which are often at considerable distance from the genes they regulate, as well as from their ability to bypass nearby, and interspersed genes. Nonetheless, mapping enhancer–target gene relationships is crucial for understanding enhancer function in both normal biology and disease. In the absence of individual experimental validation, chromatin conformation-based approaches currently offer the most effective way to predict enhancer–promoter interactions. Therefore, we utilized PR HiChIP data to link GRHL2 and/or PR sites to their target genes. While this method provides higher confidence in target assignment than assigning peaks to the nearest gene, it is not without limitations. Notably, the PR HiChIP dataset we used was generated at an early timepoint following progesterone stimulation (i.e., 30 minutes). Although this enabled the identification of the earliest GRHL2–PR co-regulated targets, additional timepoints would capture a broader dynamic range of interactions, increase the number of assignable targets, and provide insights into the temporal dynamics of PR-mediated enhancer–promoter looping. Moreover, since this dataset is PR-centered, it does not capture GRHL2-mediated chromatin loops that do not directly involve PR but may converge on the same promoters. As a result, we may be underestimating the extent of GRHL2’s regulatory influence and the full landscape of GRHL2–PR co-regulated target genes. However, separate PR and GRHL2 HiChIP datasets are also not ideal as they do not allow direct detection of PR and GRHL2 overlapping anchors. Unfortunately, a method to measure the protein directed genome architecture of two transcription factors has not been developed yet.

In summary, this study expands our knowledge on the regulatory landscape of progesterone signaling in the breast epithelium by integrating genomic, transcriptomic and proteomic data. We were able to uncover a previously unrecognized partnership between GRHL2 and PR that orchestrates hormone-responsive gene expression through dynamic chromatin binding and 3D genome organization. Our model poses that GRHL2 and PR form a complex in the absence of hormone but under these conditions, do not stably bind chromatin. Progesterone stimulation recruits GRHL2-PR complexes to enhancers followed by the formation of chromatin loops, that connect distal elements to target gene promoters. Co-binding or individual binding by GRHL2, PR, or co-factors then activates key transcriptional programs. This model provides a broad framework for understanding GRHL2–PR-mediated gene regulation. However, we find that each individual target gene ultimately is regulated by a unique combination of distal elements individually or jointly bound by GRHL2, PR and other transcription factors, which loop to its promoter. Our integrated analysis highlights the intricate and gene-specific nature of these regulatory interactions, illustrating the complexity that can arise even from the interplay of just two transcription factors.

## Limitations of the study

While our multi-omics approach provides evidence for hormone-independent physical interaction between GRHL2 and PR and their coordinated enhancer occupancy under progesterone stimulation, several limitations should be acknowledged. First, although we demonstrate co-occupancy, chromatin looping, and coordinated transcriptional regulation by PR and GRHL2, the precise molecular mechanism by which GRHL2 modulates PR-dependent enhancer activity remains unresolved. In particular, whether GRHL2 functions primarily as a pioneer factor, a scaffold for co-regulators, or a structural component contributing to loop formation as well as whether GRHL2 influences PR binding dynamics requires further mechanistic dissection. Second, most of the analysis was done in T47D(S) cells, which overexpress PR. Although this cell line is one of the very few PR responsive cell lines, which enabled controlled interrogation of GRHL2/PR crosstalk, it does not recapitulate endogenous stoichiometry or physiological regulation. Therefore, further validation of our findings in additional cell lines and breast (cancer) models will be important to assess the generality and biological relevance of the presented regulatory mechanisms. Third, since only a single well-functioning GRHL2 antibody is currently available, this antibody was used for all our experiments, including RIME, Western blots and CUT&RUN analysis, as well as in the previously generated ChIP-seq dataset that we re-analyzed. Although carefully selected and widely used, we were unable to confirm the data using alternative GRHL2 antibodies or formally exclude cross-reactivity with GRHL1. Finally, functional validation of GRHL2/PR co-regulated genes was assessed only at the mRNA level. While transcriptomic changes were robust and reproducible, protein-level validation for key targets such as IGFBP5 and TGFB2 could not be performed due to technical limitations, including the lack of suitable antibodies. Consequently, the extent to which transcriptional changes translate into altered protein abundance and downstream cellular phenotypes remains to be established.

## Materials and methods

### Cell culture

Human T47DS breast cancer cells (a kind gift from Dr. Stieneke van den Brink, Hubrecht institute, Utrecht, The Netherlands) and HEK293TN cells (System Biosciences, #LV900A-1) were routinely cultured in Dulbecco’s Modified Eagle Medium (DMEM) containing GlutaMAX (Gibco, #11584516), supplemented with 10% Fetal Bovine Serum (FBS) (Thermo Fisher Scientific, #11573397). Cells were split 1:10 every 3–4 days and routinely tested for mycoplasma. When treated, T47DS cells were cultured in DMEM containing GlutaMAX (Gibco, #11584516), supplemented with 5% Charcoal Stripped Fetal Bovine Serum (Thermo Fisher Scientific, #A3382101) for 24 hours, followed by stimulation with 1 nM R5020 (Perkin Elmer, #NLP004005MG) for the indicated time, again in stripped-serum conditions.

### Generation of T47DS GRHL2 knockdown lines

For lentiviral production, 5 × 10⁶ HEK293TN cells were seeded in 10 cm dishes. The following day, cells were transfected with 3 µg packaging vector pCMVDR8.2 (Addgene, #8455), 3 µg RSV-rev (Addgene, #12253), 3 µg VSV-G (Addgene, #12259), and 8 µg pLKO.1 GRHL2 shRNA vector (RNAi Consortium, TRCN0000015812; a kind gift from Peter Stroeken, Amsterdam UMC). After 24 hours, the medium was refreshed. Viral supernatant was collected 48 hours post-transfection, filtered through a 0.45 µm filter, and diluted 1:2. T47DS cells were infected with the viral supernatant in the presence of polybrene (1:2,000; Merck Millipore, #TR-1003-G). Following 24 hours of incubation, cells were split and selected with puromycin (1 µg/mL, Thermo Fisher Scientific, #A1113803) for up to two weeks to generate stable GRHL2 knockdown lines.

### Rapid IP-mass spectrometry of endogenous protein (RIME)

RIME analysis was performed as previously described by Mohammed *et al.* [31]. Briefly, 6x10^7^ T47DS per condition were crosslinked for 8 minutes by adding formaldehyde (Thermo Fisher Scientific, #28908) to the medium to a final concentration of 1%. The reaction was quenched by adding Glycine to a final concentration of 0.1 M. The cells were washed twice with ice-cold PBS supplemented with protease inhibitors (Sigma-Aldrich, #P8340, 1:500) and collected by scraping them off the plate. Cells were then pelleted by centrifugation 2,000 g for 3 minutes at 4 °C and lysed rotating for 5 min at 4 °C using lysis buffer LB1 (50 mM HEPES-KOH, (pH 7.5), 140 mM NaCl, 1 mM EDTA, 10% (vol/vol) glycerol, 0.5% (vol/vol) NP-40 and 0.25% (vol/vol) Triton X-100). This was followed by a second lysis in LB2 (10 mM Tris-HCL (pH 8.0), 200 mM NaCl, 1 mM EDTA and 0.5 mM EGTA), again while rotating for 5 min at 4 °C. The sample was then resuspended in LB3 (10 mM Tris-HCl (pH 8.0), 100 mM NaCl, 1 mM EDTA, 0.5 mM EGTA, 0.1% (wt/vol) sodium deoxycholate and 0.5% (vol/vol) *N*-lauroylsarcosine) and divided into three separate Eppendorf tubes for sonication in a Bioruptor pico. (Diagenode) at 4 °C using a 30 seconds on/off cycle for 10 cycles. The sonicated samples were pooled again, Triton X-100 was added to a final volume of 1% and the lysate was cleared by centrifugation. In the meantime, Dynabeads (Protein A, Invitrogen, #10001D) were washed four times in PBS containing 5 mg/ml BSA. 5 μg GRHL2 antibody (Sigma Aldrich, #HPA004820) and IgG (Santa cruz, #sc-2027X, as a negative control) were conjugated to the beads in PBS/BSA solution for 1 hour at room temperature. Sonicated nuclear lysates were pooled with antibody conjugated beads and incubated rotating at 4 °C overnight. The next day, beads were washed with RIPA buffer (50 mM HEPES (pH 7.6), 1 mM EDTA, 0.7% (wt/vol) sodium deoxycholate, 1% (vol/vol) NP-40 and 0.5 M LiCl) for a total of ten washes. For each experimental condition, three independently treated samples were processed in parallel, representing the replicates used to assess reproducibility.

From this point forward processing and analysis was performed by the Laboratory for Mass spectrometry of Biomolecules, Swammerdam Institute for life sciences, University of Amsterdam. In brief, input samples were processed as described in Hughes *et al.* [67]. Protein concentration was quantified using the Pierce BCA protein assay (Thermo Fisher Scientific, #23227), following the manufacturer’s protocol and all samples were reduced and alkylated in one step. Pull-down samples were digested on-bead according to Mohammed *et al.* [31]*.* Peptide samples were analyzed using an Ultimate 3000 RSLCnano UHPLC system (Thermo Fisher Scientific, Germeringen, Germany) coupled to a TIMS-TOF Pro mass spectrometer (Bruker, Bremen, Germany). Raw data were processed using MaxQuant (version 2.6.2.0), with trypsin/P specified as the digestion enzyme allowing up to two missed cleavages. Carbamidomethylation of cysteine was set as a fixed modification, and oxidation of methionine as a variable modification. Spectra were searched against the human Uniprot proteome database. MaxQuant IBAQ output values were used for downstream analysis using Perseus (version 2.1.3.0). Potential contaminants, proteins that were only identified by peptides that carry one or more modified amino acids, and proteins identified from a decoy sequence were filtered out. Values were Log2 transformed and rows with one missing value per experimental group were discarded. Other NaN values were replaced from normal distributed, and the three technical replicates were used to identify differentially expressed proteins between conditions using a two-sample Student’s t-test with a permutation-based false discovery rate (FDR) correction to account for multiple testing. A significance threshold of FDR < 0.05 was applied. The bait protein GRHL2 had a Log_2_fold enrichment of 3.3 and a -Log_10_(padj) of 2.2. Raw and processed RIME data are publicly available via: https://osf.io/9uqsw/.

### Co-immunoprecipitation

Co-immunoprecipitation of GRHL2 in T47DS cells was performed using approximately 3 × 10⁸ cells. Cells were washed once with PBS containing protease inhibitors (Sigma-Aldrich, #P8340, 1:500) directly on the dish, then harvested by scraping. Pelleted cells were resuspended in lysis buffer (50 mM Tris-HCl (pH 7.40), 5 mM EDTA, 0.25% Triton X-100, 10% glycerol, 100 mM NaCl) and incubated for 30 minutes at 4 °C with gentle rotation. Lysates were cleared by centrifugation at 2,000 × g for 20 minutes at 4 °C. An aliquot of the supernatant was reserved as input for the whole lysate control. The remaining lysate was incubated overnight at 4 °C with 5 µg GRHL2 antibody (Sigma-Aldrich, #HPA004820) under rotation. The following morning, 50 µL of pre-equilibrated Dynabeads Protein A (Invitrogen, #10001D) was added, and samples were rotated for 1 hour at 4 °C. Beads were washed three times with lysis buffer, and bound proteins were eluted by boiling in elution buffer (50% lysis buffer, 50% Laemmli buffer; Sigma-Aldrich, #38733) at 95 °C for 5 minutes. Eluted proteins were subsequently analyzed by western blot.

### Western blot

Western blotting was performed as previously described [68]. In short, half of the immunoprecipitated or input samples were loaded onto a 10% SDS-PAGE gel. Proteins were transferred to a 0.2 µm nitrocellulose membrane using the Trans-Blot Turbo Transfer System (Bio-Rad) and blocked with a 1:1 dilution of TBS and Odyssey Blocking Buffer (LI-COR Biosciences, #927–50100). Membranes were incubated overnight at 4 °C with primary antibodies diluted in blocking buffer supplemented with 0.1% Tween-20: anti-PR (1:1,000, Thermo Fisher Scientific, #MA5–16393, recognizing both PR-A and PR-B) and anti-GRHL2 (1:2,000, Sigma-Aldrich, #HPA004820). The following day, membranes were washed in TBS supplemented with 0.1% Tween-20 and incubated for 1 hour at RT with secondary antibodies diluted 1:20,000 in TBS with 0.1% Tween-20: IRDye 680LT (LI-COR, #926–68022) or IRDye 800CW (LI-COR, #926–32211). Signal detection was performed using the Odyssey Fc Imaging System (LI-COR Biosciences).

### RNA isolation, library preparation and RNA-seq analysis

RNA isolation, processing, and analysis for RNA-seq were performed as described previously [69]. In summary, 700,000 T47DS WT or *GRHL2* knockdown cells (stable shRNA) were seeded in 6-well plates containing DMEM + GlutaMAX (Gibco, #11584516) supplemented with 5% charcoal-stripped fetal bovine serum (Thermo Fisher Scientific, #A3382101). 24 hours after plating, the medium was refreshed, and cells were treated for an additional 24 hours with either 1 nM R5020 or ethanol (vehicle control). RNA was isolated using the RNeasy Mini Kit (Qiagen, #74104) with on-column DNase digestion (Qiagen, RNase-Free DNase Set, #79254) according to the manufacturer’s instructions.

For library preparation, 1 µg of total RNA was subjected to poly(A) selection using the NEBNext Poly(A) mRNA Magnetic Isolation Module (New England Biolabs). RNA-seq libraries were generated using the NEBNext Ultra II Directional RNA Library Prep Kit and NEBNext Multiplex Oligos for Illumina (Unique Dual Index Primer Pairs; New England Biolabs), following the manufacturer’s protocols. Library size distribution was assessed using a 2200 TapeStation System with Agilent D1000 ScreenTapes (Agilent Technologies). Quantification was performed using the NEBNext Library Quant Kit for Illumina on a QuantStudio 3 Real-Time PCR System (Thermo Fisher Scientific). Libraries were sequenced (75 bp single end) using a NextSeq 500/550 High Output Kit v2.5 (75 Cycles) on a NextSeq 550 System (Illumina). All library preparation and sequencing was carried out by MAD: Dutch Genomics Service & Support Provider, Swammerdam Institute for Life Sciences, University of Amsterdam.

Raw FASTQ files were processed on the Galaxy server. Quality control was performed using MultiQC [70], followed by trimming of overrepresented sequences with Trimmomatic [71] using a curated adapter list. Trimmed reads were aligned to the human reference genome (GRCh38.p14) using HISAT2 [72]. Mapping quality was evaluated with SAMtools stats and summarized with MultiQC. Read counts were obtained using HTSeq-count [73], and differential gene expression analysis was conducted in RStudio (version 2023.06.1) using DESeq2 [74]. The data have been deposited with NCBI GEO under accession number GSE291778. A list of Log2fold changes of the 298 GRHL2 and PR co-regulated genes and the 79 genes that are transcriptionally regulated by GRHL2 and PR and harbor a PR HiChIP loop anchor at or near their TSS are available via: https://osf.io/9uqsw/. Go term enrichment on the selected PR and GRHL2 regulated genes was performed using the *enrichGO()* function of the Clusterprofiler R package [49].

### RNA isolation and qRT-PCR

RNA isolation and qRT-PCR were performed as previously described [68]. Briefly, total RNA was extracted from T47DS cells using TRIzol Reagent (Invitrogen, #15596018) following the manufacturer’s protocol. Isolated RNA was treated with RQ1 DNase (Promega, #M6101) to remove genomic DNA contamination. cDNA synthesis was carried out using SuperScript IV Reverse Transcriptase (Invitrogen, #18090200) and Random Hexamers (Invitrogen, #N8080127), according to the manufacturer’s instructions, with the addition of RiboLock RNase Inhibitor (Thermo Fisher, #EO0328). Resulting cDNA samples were diluted 10-fold prior to qRT-PCR. Quantitative real-time PCR was performed using 5X HOT FIREPol EvaGreen qPCR Mix Plus (ROX) (Solis Biodyne, #08-24-00008) on a QuantStudio 3 Real-Time PCR System. Primer sequences are listed in Table 1. Relative expression levels were calculated using the ΔΔCt method and normalized to *YWHAZ* [75] and untreated control samples.

**Table 1:** Primers used for qRT-PCR.

Gene	Forward (5’-3’)	Reverse (5’-3’)
*GRHL2*	CAAAGCAAGTGACAGCCAAG	CTTTGTTGAGGTAGGTCATGG
*TGFB2*	ACACTCAGCACAGCAGGGTCCT	ACACTCAGCACAGCAGGGTCCT
*IGFB5*	CGGGGTTTGCCTCAACGAA	TCTTGGGGGAGTAGGTCTCCT
*YWHAZ*	ACTTTTGGTACATTGTGGCTTCAA	CCGCCAGGACAAACCAGTAT

### CUT&RUN Low Volume Urea (LoV-U)

CUT&RUN LoV-U was performed as described in Zambanini *et al*. [76], with the following modifications. Approximately 500,000 T47DS cells per sample were harvested using 0.25% trypsin, and cells were washed three times in nuclear extraction buffer (20 mM HEPES-KOH (pH 8.2), 10 mM KCl, 0.5 mM spermidine, 0.05% IGEPAL, 20% glycerol, and Roche Complete Protease Inhibitor EDTA-Free) to isolate nuclei. Nucleic pellets were flash-frozen in an isopropyl chamber and stored at –80 °C until further processing. Nuclei were thawed, washed once in nuclear extraction buffer and subsequently bound to equilibrated magnetic ConA agarose beads (Antibodies-online, #ABIN6952467). Following incubation, nuclei-bead complexes were washed for 5 minutes in wash buffer (20 mM HEPES (pH 7.5), 150 mM NaCl, 0.5 mM spermidine, and Roche Complete Protease Inhibitor EDTA-Free, 0.025% digitonin) supplemented with 2 mM EDTA, and then transferred to PCR tubes. Antibodies (anti-PR (1:100, Cell Signaling, #8757S), anti-GRHL2 (1:100, Sigma-Aldrich, #HPA004820), or rabbit IgG isotype control (1:100, Invitrogen, #100500C)) were added to the wash buffer and incubated overnight at 4 °C on a rotator. The next day, samples were washed five times with wash buffer, then resuspended in pAG-MN buffer (wash buffer containing 120 ng/sample pAG-MN) and incubated for 30 minutes at 4 °C on a rotator. Following another five washes, digestion was initiated by resuspending the beads in wash buffer supplemented with 2 mM CaCl₂ and incubated at room temperature for 5 minutes, followed by an additional 25 minutes at 4 °C. The reaction was stopped by adding 250 mM EDTA/EGTA mix. Fragment release was initiated by the addition of 5 M NaCl and incubation at 37 °C for 30 minutes. The supernatant was collected, and beads were subsequently resuspended in 1 × Urea buffer (100 mM NaCl, 2 mM EDTA, 2 mM EGTA, 0.5% IGEPAL, 8.5 M urea) for a second release step. After 30 minutes at room temperature, the urea release was combined with the initial supernatant. Samples were treated with SDS and proteinase K, followed by phenol:chloroform:isoamyl alcohol extraction and ethanol precipitation. Libraries were sequenced on an Illumina NextSeq 550 platform using 36 bp paired-end reads to a depth of 5–15 million reads per sample.

### CUT&RUN data analysis

Quality of the reads was assessed using fastqc [77]. Reads were trimmed with bbmap bbdud [78] to remove adapters and poly [AT], [G] and [C] repeat sequences. Reads were aligned to the hg38 genome with bowtie2 [79] with the options –local –very-sensitive-local –no-unal –no-mixed -no-discordant –phred33 –dovetail -I 0 -X 500. Samtools suite [80] was used to remove duplicate and incorrectly paired reads. Bedtools [81] was used to remove reads mapped to the CUT&RUN hg38 suspect list [82] from bam files. Bedgraphs were created using Bedtools genomecov on pair-end mode. Peaks were called using MACS2 [83] with the options -f BAMPE and -p 1e-3 against the IgG control for narrowPeaks. The CUT&RUN data have been deposited with NCBI GEO under accession number GSE307203.

### Re-analysis of ChIP-seq datasets

Raw ChIP-seq datafiles from PR [38] (Control; SRX11374768, and 1 nM R5020 treated; SRX11374766) and GRHL2 [23] (two replicates; SRX5350536, SRX5350537) datasets were downloaded from the SRA server. Quality of the reads was assessed using fastqc [77] (version 0.11.9), followed by trimming of overrepresented sequences adapters and PCR primers with Trimmomatic [84]. Trimmed reads were aligned to the hg38 genome with bowtie2 [79] with the options –local –very-sensitive-local –no-unal –no-mixed -no-discordant – dovetail -I 0 -X 500. Samtools [80] was used to convert sam to bam files, fix improperly paired mates, and remove duplicates. Bedtools [81] was used to remove reads mapped to the hg38 blacklisted regions [85]. Final read counts of usable fragments were determined from filtered and deduplicated bam files. Bedgraphs were created with Bedtools genomecov on pair-end mode. Bedgraps were visualized in IGV [39]. For peak calling, visualization and signal graphs, replicate bam files were merged with SAMtools into a single file. Peaks were called using MACS2 [83] with the options -f BAMPE and -p 1e-3 for narrowPeaks. deepTools [86] was used to convert bam files to normalized BigWig files (bamCoverage using -RPGC option and -e to extend reads to fragment length). Bigwigs were visualized in IGV; tracks shown in the figures are a merge of two biological replicates (GRHL2) or single replicate (PR), normalized to reads per genome coverage, and scaled by factor.

### Downstream analysis

For both ChIP-seq and CUT&RUN signal intensity heatmaps and signal profiles were generated using deepTools [86]. Peak overlaps were calculated using Bedtools [81], requiring a minimum of 1 bp overlap. Venn diagrams were created with the *Venndiagram* R package, and genome annotation was performed using the *ChIPseeker* [40,41] R package. Motif enrichment analysis was carried out using HOMER [44] with the options -size 200 and -mask. Processed PR HiChIP data from R5020-stimulated T47D cells (Zaurin *et al*. [38], GEO accession: GSM5425945) were downloaded in.bedpe format and overlapped with TSS regions (–5 kb to +1 kb from the TSS) of 549 genes identified as PR- and GRHL2-regulated by RNA-seq, using Bedtools pairtobed [81]. TSS regions containing a HiChIP anchor were selected for further analysis. Peak-called ChIP-seq datasets for R5020-stimulated H3K27ac [42] (SRX22674023) RAD21 [42] (SRX22674036) and CTCF [42] (SRX22674025) as well as ATAC-seq datasets [43] for siControl (SRX23397061) and siGRHL2 (SRX23397063) were retrieved from ChIPatlas [87].

### Clinical data analysis

Gene expression and clinical data for primary breast tumors were obtained from the METABRIC (Molecular Taxonomy of Breast Cancer International Consortium) cohort via cBioPortal [45–48]. Probes without gene annotations were removed, and for genes with multiple probes, the probe with the highest variance across samples was retained. Clinical data from both sample- and patient-level files were merged. Only tumors with both clinical and expression data were included, resulting in a final dataset of 1,980 patients for survival analyses. The 298 genes co-regulated by GRHL2 and PR (as determined in Fig 4) were merged into a gene signature and the *Hacksig* [88] R package was used to calculate a composite signature score for each tumor signature. Up- and down-regulated genes were scored separately and combined to yield a total signature score. The scores were standardized (z-score) across patients, and high- and low-signature groups were defined using the median as a cutoff. Expression levels of *PGR GRHL2*, were extracted from the expression matrix for downstream analyses and boxplots were generated to compare gene and signature expression between PR-positive and PR-negative tumors. Statistical significance was assessed using Wilcoxon rank-sum tests. Survival data was analysised using the *Survival* [89,90] and *Survminer* [91] R packages. Kaplan–Meier survival curves were generated for high- versus low-signature tumors in the full cohort. Differences in survival were assessed using the log-rank test. Multivariable Cox proportional hazards regression models were used to evaluate the association between the GRHL2/PR signature score and overall survival, adjusting for age at diagnosis and tumor stage. Interaction models were also tested to assess whether the prognostic impact of the signature differed between PR-positive and PR-negative tumors.

## Supporting information

S1 Fig R5020-induced GRHL2 binding sites identified by CUT&RUN align with GRHL2 ChIP-seq profiles. A) Top five transcription factor motifs that were determined as enriched in all 1 nM R5020 stimulated PR peaks, all non-treated GRHL2 peaks or the GRHL2-PR shared peaks as determined by HOMER motif analysis [44]. B) Venn diagram showing the overlap between 4 hour 1 nm R5050 stimulated GRHL2 and PR CUT&RUN peaks. Peaks were considered overlapping if they overlapped by at least 1 bp. C) A representative example of two PR and GRHL2 overlapping CUT&RUN peaks. Data was visualized in the IGV browser [39]. NT = non-treated. D) Heatmaps showing CUT&RUN signal of PR R5020 treated and GRHL2 in untreated and 4 hour 1 nM R5020 stimulated conditions, at PR-only, GRHL2-only and GRHL2-PR shared ChIP-seq peaks.
(TIF)

S2 Fig Validation of direct GRHL2 – PR target genes. A) Western blot for GRHL2 of wildtype (WT) and shRNA mediated *GRHL2* knockdown (shGRHL2) T47DS cells stimulated with 1 nM R5020 for 4 hours. Three independent biological replicates are shown. ACTIN was used as a loading control. B) Bar graph showing the relative GRHL2 protein expression over ACTIN of the western blot in (A). Individual values are normalized over the non-treated WT (control). Datapoints: individual values for n = 3 biological replicates depicted as mean fold change normalized to control. P-values were calculated using a two-way ANOVA followed by a Uncorrected Fisher’s Least Significant Difference test. C) Principal component analysis (PCA) of all genes expressed in the bulk RNA-seq in WT and shGRHL2 T47DS cells that were non-treated (NT) or stimulated for 24 hours with 1 nM R5020. Each dot represents one replicate; each group contains n = 3 replicates.
(TIF)

S3 Fig The high GRHL2/PR regulated gene expression is associated with reduced overall survival of breast cancer patients. A-B) Boxplots showing the distribution of *PGR* (A) and *GRHL2* (B) mRNA expression (log2-normalized) stratified by PR status in the METABRIC cohort. Individual tumors are shown as jittered points. C) Kaplan–Meier analysis of METABRIC tumors stratified into high- and low-signature groups based on the median GRHL2/PR signature score. Patients with high signature expression (red) exhibited significantly worse overall survival compared to those with low expression (blue) (log-rank test, P < 0.001). D) Boxplots showing the distribution of GRHL2/PR signature scores stratified by PR status in the METABRIC cohort. Individual tumors are shown as jittered points.
(TIF)

S4 Fig GRHL2- or PR bound enhancers can converge through 3D chromatin looping to jointly regulate gene expression. A) Density plot of the loop size distribution of the 146 PR HiChIP [38] loops that are directly link to a GRHL2/PR regulated TSS as determined by RNA-seq. Loop size is displayed in kilobases (kb). B) Selection of transcription factor motifs that were determined as enriched at anchors that did not harbor a GRHL2 peak, PR peak, or regulated TSS, were defined as ‘Other’. Motif enrichment analysis was performed using HOMER [44]. C) Visual representation of individual non-overlapping GRHL2 and PR peaks, and the *ARID5B* promotor that converge on one PR peak by chromatin looping, showing PR ChIP-seq [38] peaks, GRHL2 ChIP-seq [23] peaks and PR HiChIP loops [38]. Grey bar highlights the *ARID5B* TSS. Orange bars highlight the individual PR and GRHL2 peaks. Data was visualized in the IGV browser [39].
(TIF)

S5 Fig GRHL2 interacts with Cohesin complex members. A) Volcano plot depicting the results of the GRHL2 RIME of hormone depleted conditions vs. the IgG control. Each grey dot represents a single protein identified in mass spectrometry. Grey dotted lines represent Log2FC (-3,3) and -Log10(p-value) cutoffs (0.05) 2,325 proteins passed these criteria and were identified as a GRHL2 interactor. Highlighted dots in red are known Cohesin subunits, loading and releasing factors that are significantly interacting with GRHL2. B-C) Venn diagrams showing the overlap between non-treated and 30 minutes R5020 treated CTCF ChIP-seq peaks [42] (B) or between non-treated and 30 minutes R5020 treated RAD21 ChIP-seq peaks [42] (C) in T47D cells. Reanalysis of data (i.e., called peaks) from ChIP-Atlas. Peaks were considered overlapping if they overlapped by at least 1 bp.
(TIF)
