# Supplementary figures and images for: Mechanistic dissection of GRHL2 and PR transcriptional co-regulation in breast cells
Source: PLoS Genet. 2026 Mar 17;22(3):e1012088. doi: 10.1371/journal.pgen.1012088 (PMC13008249; doi:10.1371/journal.pgen.1012088)

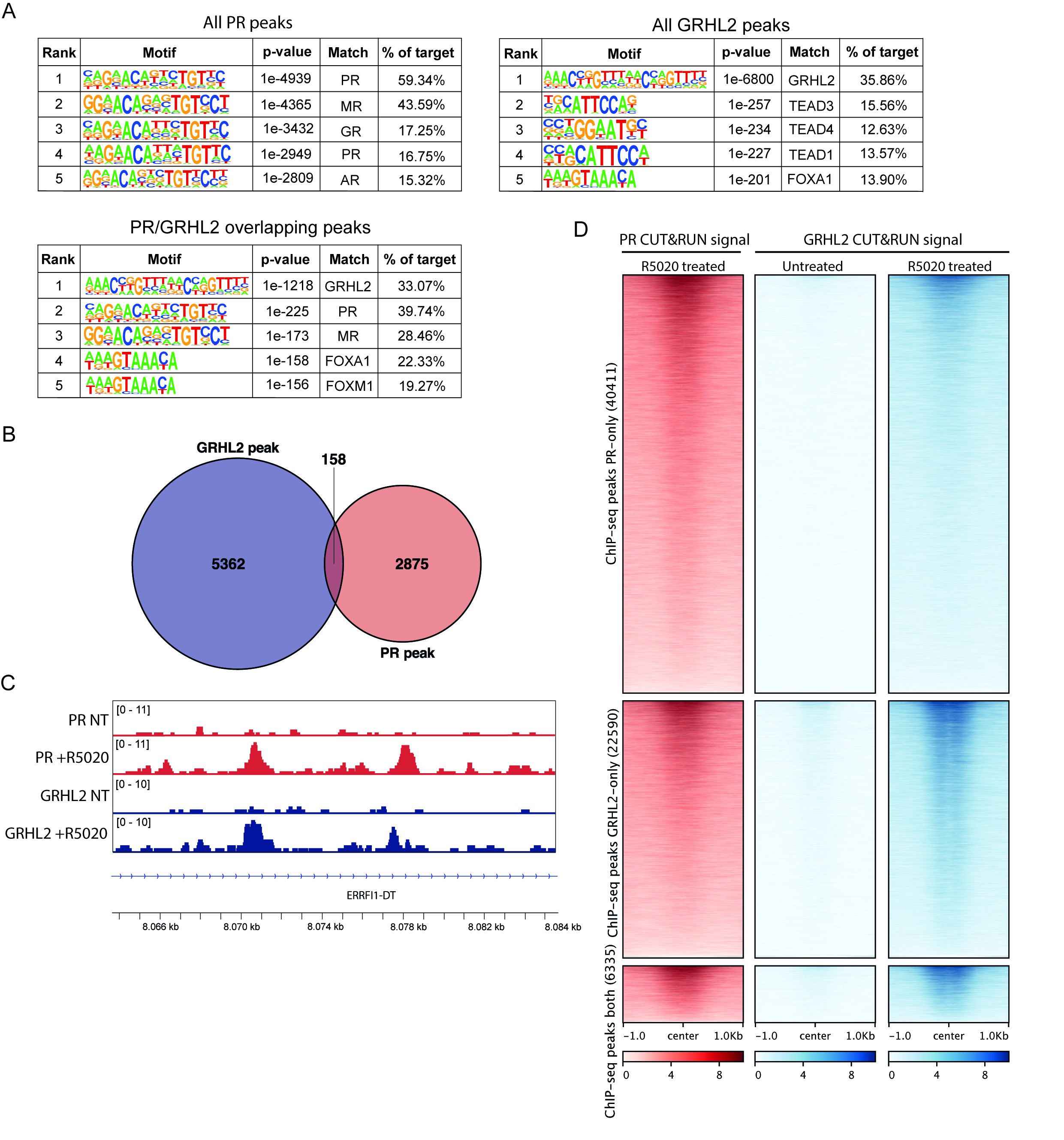

Supplement: S1 Fig — A) Top five transcription factor motifs that were determined as enriched in all 1 nM R5020 stimulated PR peaks, all non-treated GRHL2 peaks or the GRHL2-PR shared peaks as determined by HOMER motif analysis [44]. B) Venn diagram showing the overlap between 4 hour 1 nm R5050 stimulated GRHL2 and PR CUT&RUN peaks. Peaks were considered overlapping if they overlapped by at least 1 bp. C) A representative example of two PR and GRHL2 overlapping CUT&RUN peaks. Data was visualized in the IGV browser [39]. NT = non-treated. D) Heatmaps showing CUT&RUN signal of PR R5020 treated and GRHL2 in untreated and 4 hour 1 nM R5020 stimulated conditions, at PR-only, GRHL2-only and GRHL2-PR shared ChIP-seq peaks. (TIF) [file pgen.1012088.s001.tif]

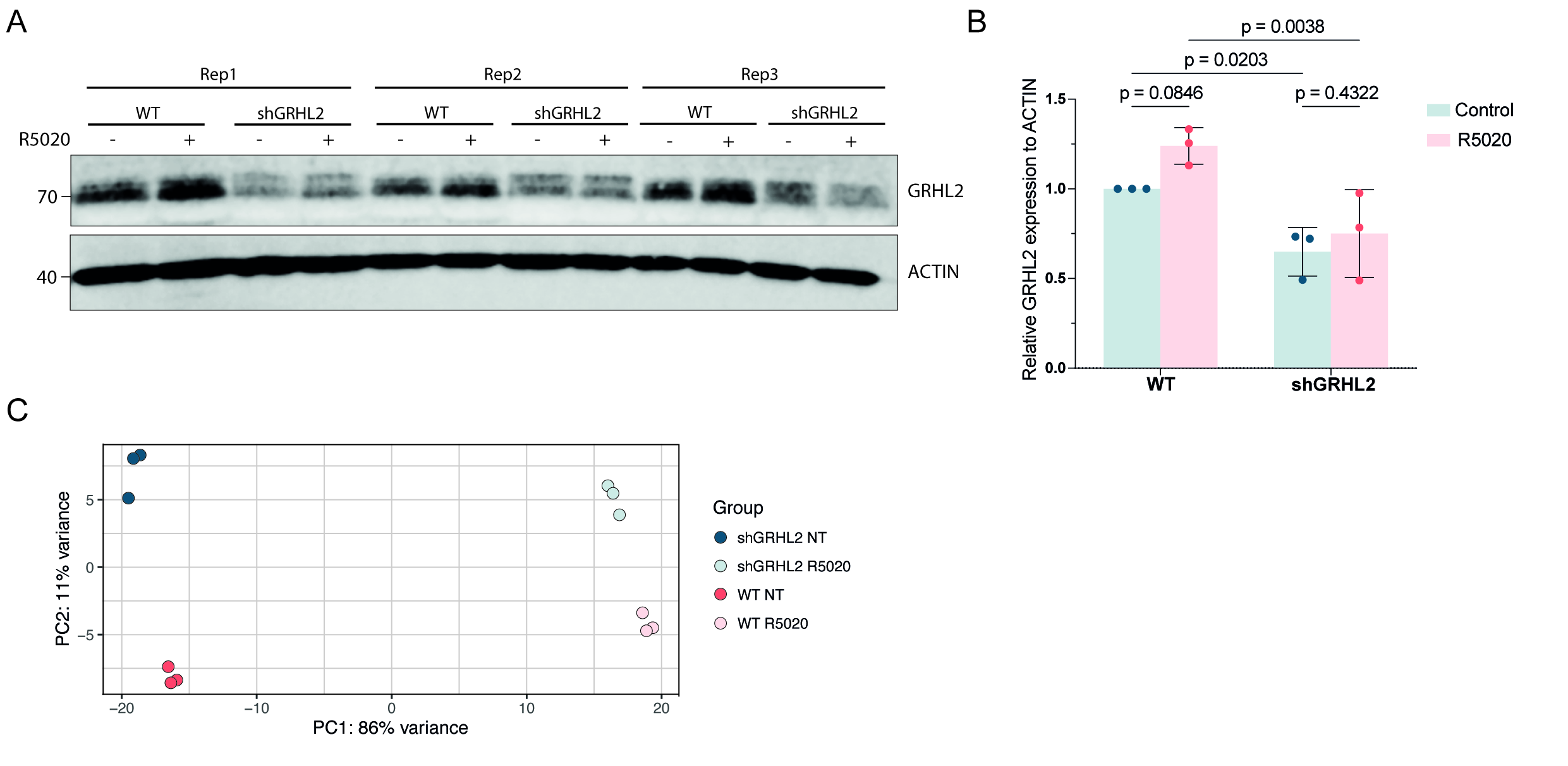

Supplement: S2 Fig — A) Western blot for GRHL2 of wildtype (WT) and shRNA mediated GRHL2 knockdown (shGRHL2) T47DS cells stimulated with 1 nM R5020 for 4 hours. Three independent biological replicates are shown. ACTIN was used as a loading control. B) Bar graph showing the relative GRHL2 protein expression over ACTIN of the western blot in (A). Individual values are normalized over the non-treated WT (control). Datapoints: individual values for n = 3 biological replicates depicted as mean fold change normalized to control. P-values were calculated using a two-way ANOVA followed by a Uncorrected Fisher’s Least Significant Difference test. C) Principal component analysis (PCA) of all genes expressed in the bulk RNA-seq in WT and shGRHL2 T47DS cells that were non-treated (NT) or stimulated for 24 hours with 1 nM R5020. Each dot represents one replicate; each group contains n = 3 replicates. (TIF) [file pgen.1012088.s002.tif]

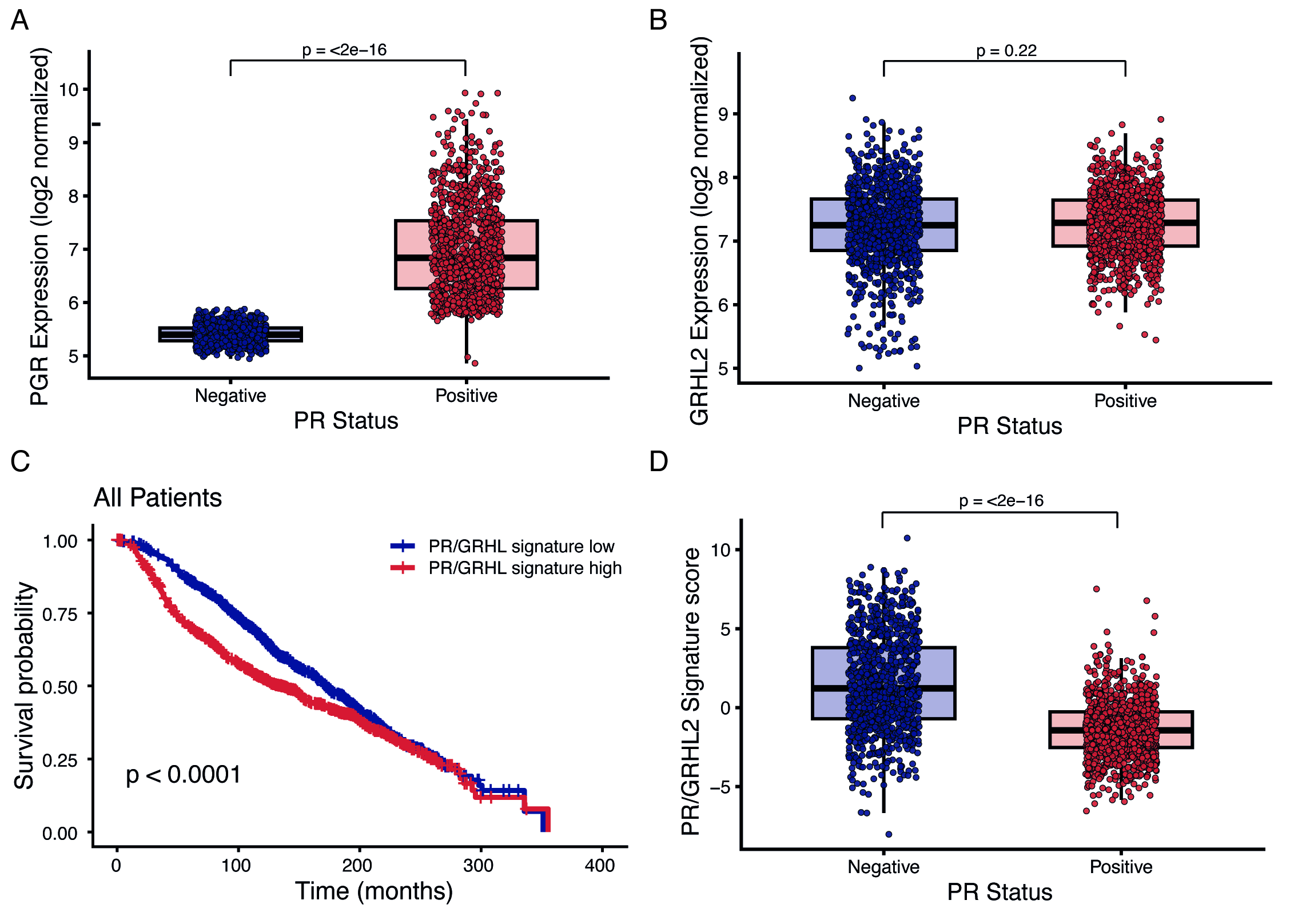

Supplement: S3 Fig — A-B) Boxplots showing the distribution of PGR (A) and GRHL2 (B) mRNA expression (log2-normalized) stratified by PR status in the METABRIC cohort. Individual tumors are shown as jittered points. C) Kaplan–Meier analysis of METABRIC tumors stratified into high- and low-signature groups based on the median GRHL2/PR signature score. Patients with high signature expression (red) exhibited significantly worse overall survival compared to those with low expression (blue) (log-rank test, P < 0.001). D) Boxplots showing the distribution of GRHL2/PR signature scores stratified by PR status in the METABRIC cohort. Individual tumors are shown as jittered points. (TIF) [file pgen.1012088.s003.tif]

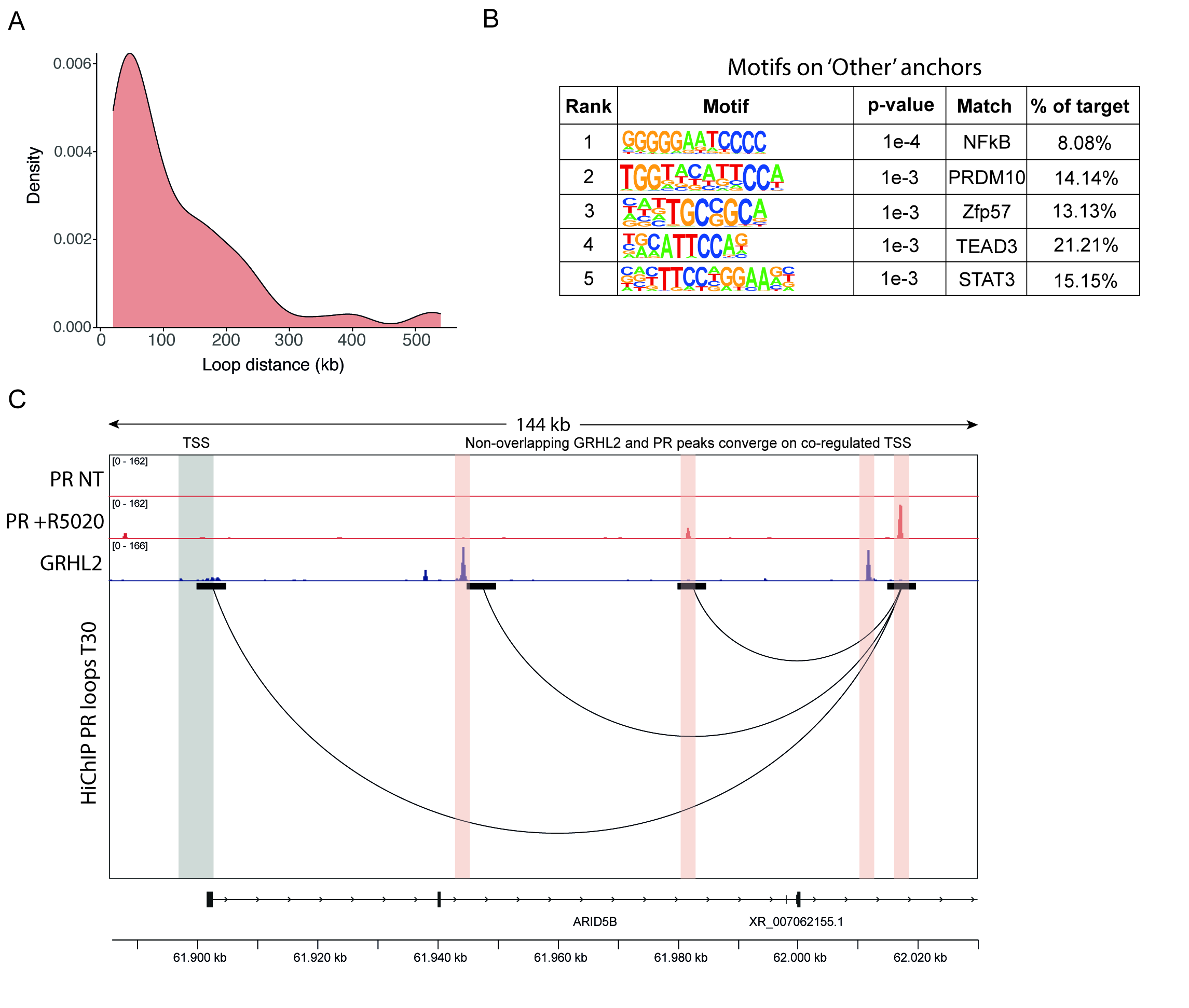

Supplement: S4 Fig — A) Density plot of the loop size distribution of the 146 PR HiChIP [38] loops that are directly link to a GRHL2/PR regulated TSS as determined by RNA-seq. Loop size is displayed in kilobases (kb). B) Selection of transcription factor motifs that were determined as enriched at anchors that did not harbor a GRHL2 peak, PR peak, or regulated TSS, were defined as ‘Other’. Motif enrichment analysis was performed using HOMER [44]. C) Visual representation of individual non-overlapping GRHL2 and PR peaks, and the ARID5B promotor that converge on one PR peak by chromatin looping, showing PR ChIP-seq [38] peaks, GRHL2 ChIP-seq [23] peaks and PR HiChIP loops [38]. Grey bar highlights the ARID5B TSS. Orange bars highlight the individual PR and GRHL2 peaks. Data was visualized in the IGV browser [39]. (TIF) [file pgen.1012088.s004.tif]

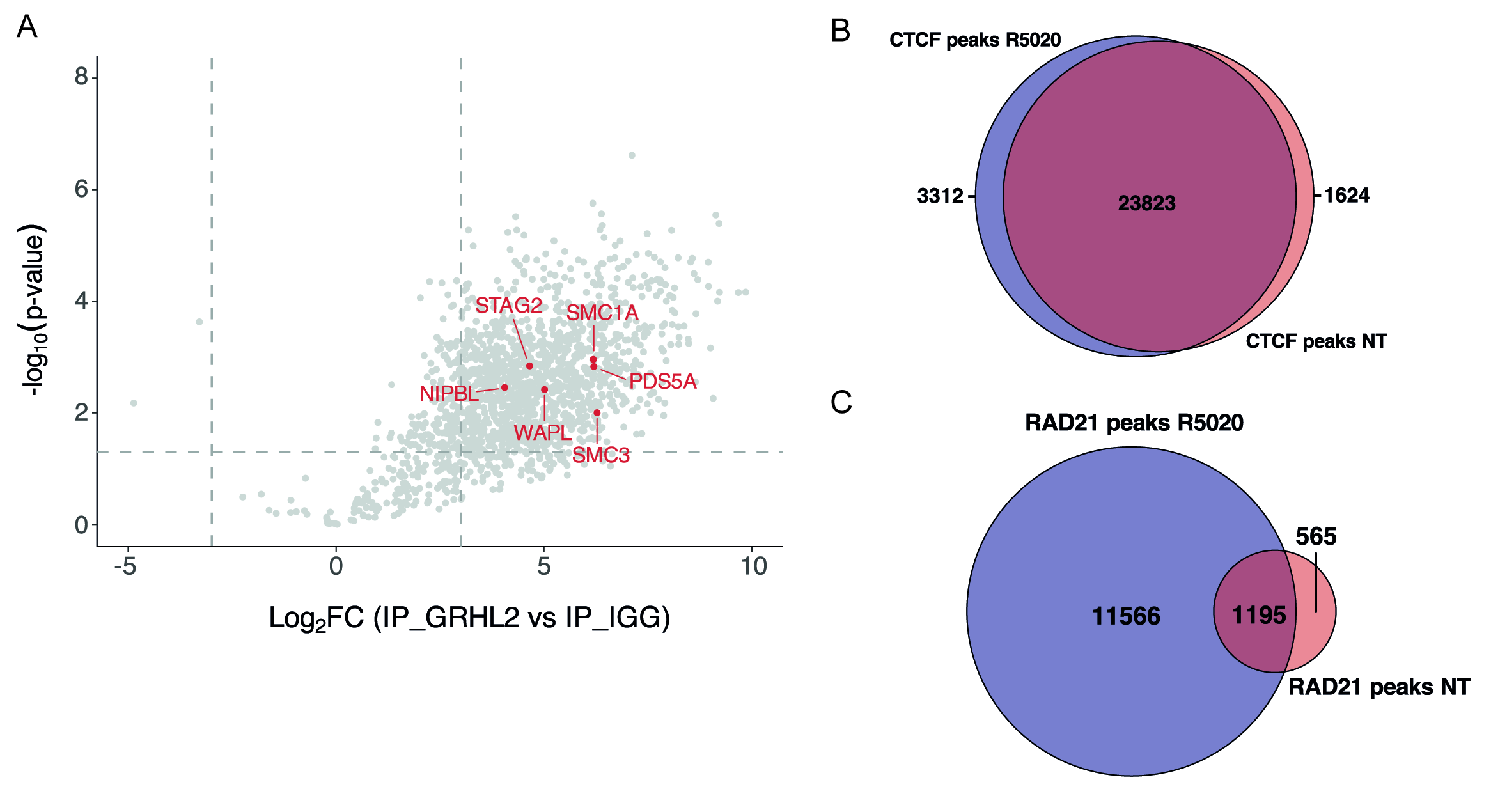

Supplement: S5 Fig — A) Volcano plot depicting the results of the GRHL2 RIME of hormone depleted conditions vs. the IgG control. Each grey dot represents a single protein identified in mass spectrometry. Grey dotted lines represent Log2FC (-3,3) and -Log10(p-value) cutoffs (0.05) 2,325 proteins passed these criteria and were identified as a GRHL2 interactor. Highlighted dots in red are known Cohesin subunits, loading and releasing factors that are significantly interacting with GRHL2. B-C) Venn diagrams showing the overlap between non-treated and 30 minutes R5020 treated CTCF ChIP-seq peaks [42] (B) or between non-treated and 30 minutes R5020 treated RAD21 ChIP-seq peaks [42] (C) in T47D cells. Reanalysis of data (i.e., called peaks) from ChIP-Atlas. Peaks were considered overlapping if they overlapped by at least 1 bp. (TIF) [file pgen.1012088.s005.tif]
